# Effects of enhanced recovery after surgery and prehabilitation interventions on postoperative outcomes after lung resection in patients with lung cancer: a systematic review and meta-analysis

**DOI:** 10.3389/fpubh.2026.1939340

**Published:** 2026-10-05

**Authors:** Dongjin Kim, Gisoo Shin

**Affiliations:** 1 Department of Nursing, Seoul National University Bundang Hospital, Gyeonggi-do, Republic of Korea; 2 College of Nursing, Chung-Ang University, Seoul, Republic of Korea

**Keywords:** enhanced recovery after surgery, lung cancer, meta analysis, patient-reported outcomes, prehabilitation, systematic review

## Abstract

**Introduction:**

Lung resection is the standard curative treatment for resectable lung cancer; however, postoperative pulmonary complications (PPCs) and prolonged hospital length of stay (LOS) remain major barriers to recovery and healthcare efficiency. Enhanced recovery after surgery (ERAS) and prehabilitation are distinct perioperative strategies that differ in their timing, intervention components, and mechanisms of action, but both aim to optimize patients’ perioperative condition and improve postoperative recovery. However, their effectiveness compared with conventional perioperative care based solely on randomized controlled trials (RCTs) has not been comprehensively synthesized.

**Objectives:**

To assess the effectiveness of ERAS and prehabilitation in reducing PPCs, shortening LOS, and improving patient-reported outcomes following pulmonary resection for lung cancer.

**Subjects and methods:**

We conducted a systematic review and meta-analysis of RCTs involving adults undergoing pulmonary resection for primary lung cancer. Searches of PubMed, Embase, CENTRAL, CINAHL Complete, RISS, KISS, ClinicalTrials.gov, and the WHO International Clinical Trials Registry Platform identified studies published between January 2015 and May 2026. Risk of bias was assessed using the Cochrane Risk of Bias 2 tool, and certainty of evidence was evaluated using GRADE. Random-effects meta-analyses estimated pooled odds ratios (ORs) for PPCs and mean differences (MDs) for LOS. Patient-reported outcomes were synthesized narratively according to the SWiM guideline.

**Results:**

Ten RCTs involving 1,569 participants met the inclusion criteria. ERAS and prehabilitation significantly reduced PPCs compared with conventional perioperative care (OR = 0.48, 95% CI = 0.36–0.65, *p* < 0.001). The exploratory pooled analysis suggested a shorter postoperative LOS (MD = −2.30 days, 95% CI = −3.58 to −1.01); however, heterogeneity was considerable (*I*^2^ = 97.9%), and the 95% prediction interval crossed the null. Narrative synthesis demonstrated consistent improvements in anxiety, depression, postoperative pain, pulmonary function, quality of life, and functional recovery.

**Conclusion:**

ERAS and prehabilitation are effective perioperative strategies for improving outcomes after pulmonary resection for lung cancer. Their implementation in routine clinical practice may reduce postoperative complications, accelerate recovery, and improve healthcare efficiency. Further multicenter RCTs are needed to optimize intervention components, standardize outcome measures, and strengthen evidence for long-term and patient-reported outcomes.

**Systematic review registration:**

CRD420261373242.

## Introduction

Lung cancer remains one of the leading causes of cancer incidence and mortality worldwide, including in South Korea (1, 2). According to GLOBOCAN 2022, approximately 2.48 million new cases of lung cancer and 1.80 million lung cancer–related deaths were reported globally, making lung cancer the leading cause of cancer mortality worldwide (3). Approximately 80–85% of lung cancers are classified as non-small cell lung cancer (NSCLC), for which pulmonary resection remains the standard curative treatment for patients with resectable early-stage disease (stages I–II) and selected patients with locally advanced disease (stage IIIA) (2). Although minimally invasive surgical approaches, including video-assisted thoracoscopic surgery (VATS) and robot-assisted thoracic surgery (RATS), have substantially improved perioperative outcomes, pulmonary resection remains an invasive procedure involving the removal of functional lung parenchyma and is therefore associated with considerable postoperative morbidity (4).

Among postoperative complications, postoperative pulmonary complications (PPCs) represent one of the most clinically significant adverse outcomes following pulmonary resection (5). PPCs encompass a broad spectrum of respiratory complications, including atelectasis, pneumonia, bronchospasm, respiratory failure, acute respiratory distress syndrome, and prolonged air leakage, all of which adversely affect postoperative recovery and long-term prognosis (6). Recent meta-analyses have reported PPC incidences ranging from 9 to 37%, with a pooled incidence of approximately 18%. Patients who develop PPCs experience prolonged hospitalization, increased healthcare costs, and substantially higher risks of severe complications and mortality than those without PPCs (7). Consequently, preventing PPCs has become a major objective of perioperative management for patients undergoing lung resection (6, 7).

Length of hospital stay (LOS) is another important indicator of postoperative recovery following pulmonary resection. In addition to reflecting functional recovery, LOS represents healthcare resource utilization and economic burden and is strongly associated with the occurrence of PPCs (8). Although LOS may be influenced by multiple factors, including surgical approach, chest tube management, discharge criteria, and healthcare delivery systems, it remains one of the most widely accepted outcome measures for evaluating the effectiveness of perioperative interventions (9). Therefore, effective perioperative strategies that simultaneously reduce PPCs and shorten LOS are essential for optimizing postoperative recovery.

Beyond physical recovery, patients undergoing pulmonary resection frequently experience substantial psychological and social challenges. Postoperative anxiety, depression, pain, and impaired quality of life may negatively affect treatment adherence, functional recovery, and long-term outcomes (10). Accordingly, increasing attention has been directed toward incorporating patient-reported outcomes (PROs) alongside conventional clinical endpoints when evaluating perioperative interventions (11). This shift highlights the importance of comprehensive perioperative care aimed not only at reducing postoperative complications but also at improving patients’ overall physical and psychological recovery (12).

To enhance postoperative recovery and reduce surgical complications, several perioperative interventions have been developed. Among these, enhanced recovery after surgery (ERAS) programs and prehabilitation have emerged as two evidence-based strategies with growing clinical acceptance (13). Although both interventions share the common goal of improving postoperative recovery, they differ in their timing and primary focus.

ERAS is a multidisciplinary perioperative care pathway that standardizes evidence-based practices throughout the preoperative, intraoperative, and postoperative phases of surgical care (13, 14). In thoracic surgery, current guidelines from the ERAS Society and the European society of thoracic surgeons (ESTS) recommend integrated implementation of preoperative patient education, nutritional optimization, smoking cessation, respiratory exercises, lung-protective ventilation, multimodal analgesia, early oral feeding, early mobilization, and standardized chest tube management (15). Collectively, these multimodal interventions aim to attenuate the surgical stress response, reduce postoperative complications, and facilitate functional recovery (13–15).

In contrast, prehabilitation focuses on optimizing patients’ physiological reserve during the preoperative period before surgical stress occurs (16). Prehabilitation programs typically combine aerobic and resistance exercise training, inspiratory muscle training, nutritional support, and psychological interventions to improve functional capacity and enhance resilience to surgery (17). Previous studies have suggested that prehabilitation may be particularly beneficial for high-risk patients, including older adults and those with chronic obstructive pulmonary disease (COPD) or poor baseline exercise capacity (18).

Recent systematic reviews and meta-analyses have demonstrated that both ERAS and prehabilitation reduce postoperative pulmonary complications and shorten hospital stay after pulmonary resection (16–18). Some studies have also suggested improvements in exercise capacity, quality of life, anxiety, and depression. However, the available evidence remains heterogeneous because intervention components, intensity, duration, and outcome assessment vary substantially across studies (19). Furthermore, many previous reviews have included retrospective studies or small clinical trials, limiting the overall certainty of the evidence. Importantly, ERAS and prehabilitation represent distinct approaches to perioperative optimization. ERAS comprises multimodal interventions implemented across the perioperative pathway, whereas prehabilitation primarily focuses on enhancing patients’ physiological and psychological reserve before surgery. Despite these differences in timing, intervention components, and mechanisms of action, both strategies share the broader clinical goal of improving patients’ preparedness for and recovery from surgical stress (20).

Accordingly, the present systematic review and meta-analysis aimed to comprehensively evaluate the effectiveness of ERAS and prehabilitation compared with conventional perioperative care among patients undergoing pulmonary resection for lung cancer, based exclusively on randomized controlled trials. Importantly, because no head-to-head randomized trials directly comparing ERAS with prehabilitation were included, the present review was not intended to establish the comparative effectiveness or equivalence of these two intervention strategies.

## Methods

### Study design

This systematic review and meta-analysis was conducted in accordance with the Preferred Reporting Items for Systematic Reviews and Meta-Analyses (PRISMA) 2020 statement. The review protocol was prospectively registered in the International Prospective Register of Systematic Reviews (PROSPERO) (Registration No. CRD420261373242). Before study selection and data extraction, the intervention categories (ERAS and prehabilitation) and the primary outcomes, including PPCs, LOS, and PROs, were predefined in the study protocol.

ERAS and prehabilitation were treated as distinct intervention categories within a broader framework of multimodal perioperative optimization. An overall synthesis was conducted because both strategies were evaluated against conventional perioperative care and shared the overarching goal of improving perioperative preparedness and postoperative recovery. To account for their differences in timing, intervention components, and mechanisms of action, intervention type (ERAS vs. prehabilitation) was prespecified for subgroup analysis. Accordingly, the overall pooled analysis was intended to estimate the average effect of multimodal perioperative optimization strategies compared with conventional perioperative care, rather than to directly compare ERAS with prehabilitation.

### Eligibility criteria

Study eligibility was determined according to the PICOS (participants, intervention, comparator, outcomes, and study design) framework. The eligibility criteria were prespecified in the registered review protocol.

Studies were excluded if they: included patients undergoing pulmonary resection for diseases other than primary lung cancer (e.g., benign pulmonary lesions, pulmonary tuberculosis, or pulmonary metastases from extrapulmonary malignancies); included patients receiving chemotherapy or radiotherapy without surgical resection; enrolled pediatric patients (<18 years of age); investigated lung transplantation, lung volume reduction surgery, or isolated pleural procedures without pulmonary parenchymal resection; included mixed disease populations in which patients with lung cancer accounted for <80% of the study population or when lung cancer–specific data could not be extracted separately; evaluated single-component interventions rather than multimodal ERAS or prehabilitation programs; used active comparator interventions rather than conventional perioperative care; were published in a language other than English or Korean; were published before January 2015; or were quasi-randomized trials, observational studies, case reports, case series, review articles, or previous systematic reviews.

### Literature search

A comprehensive literature search was performed in PubMed (MEDLINE), Embase, the Cochrane Central Register of Controlled Trials (CENTRAL), CINAHL Complete, RISS, KISS, ClinicalTrials.gov, and the World Health Organization International Clinical Trials Registry Platform (WHO ICTRP).

The search covered publications from January 2015 to May 2026. This window was prespecified in the registered protocol so as to span the period both before and after publication of the joint ERAS Society and European Society of Thoracic Surgeons guideline for enhanced recovery after lung surgery, which established the reference standard for contemporary multimodal perioperative protocols. Searches were restricted to studies published in English or Korean, as prespecified in the registered protocol. Language filters were applied at the database level (English and Korean for PubMed, Embase and CENTRAL; English for CINAHL Complete; Korean for RISS and KISS). Search strategies were developed according to the PICOS framework. Controlled vocabulary (MeSH and Emtree terms) and free-text keywords were combined using Boolean operators. Search terms included concepts related to lung cancer (“lung cancer,” “non-small cell lung cancer,” “pulmonary resection,” and “lung resection”) and perioperative interventions (“enhanced recovery after surgery,” “ERAS,” “fast-track surgery,” and “prehabilitation”). Searches were limited to human studies.

To minimize publication bias, gray literature was identified through ClinicalTrials.gov, WHO ICTRP, RISS, and KISS. In addition, backward citation searching of eligible studies and relevant systematic reviews was performed to identify additional studies.

### Study selection

Study selection was independently conducted by two reviewers using the blinded review mode of Rayyan. Before screening, reviewers underwent calibration exercises using pilot studies to ensure consistent application of the eligibility criteria.

Titles and abstracts were screened during the first stage, followed by full-text assessment of potentially eligible articles. Disagreements were resolved through discussion, and when necessary, consultation with a third reviewer. Inter-reviewer agreement was evaluated using Cohen’s kappa coefficient (κ = 0.66 on the 50-record calibration sample, indicating substantial agreement).

The study selection process is presented using the PRISMA 2020 flow diagram.

### Data extraction

Data were independently extracted by two reviewers using a standardized Microsoft Excel data extraction form developed before the review.

The following information was collected: study characteristics (author, publication year, country, and study design); participant characteristics; intervention characteristics (type, components, timing, and healthcare provider); comparator characteristics; outcome measures, assessment tools, follow-up periods, and attrition rates; statistical data required for meta-analysis.

When standard deviations were unavailable, they were estimated from reported 95% confidence intervals or interquartile ranges according to the recommendations of the Cochrane Handbook (Version 6.5.2). Data reported as medians and interquartile ranges were converted into means and standard deviations using the method proposed by Wan et al. (21).

### Risk of bias assessment

The methodological quality of included randomized controlled trials was independently assessed by two reviewers using the Cochrane Risk of Bias 2 (RoB 2) tool. Risk of bias was assessed at the outcome level for the two primary outcomes (postoperative pulmonary complications and length of hospital stay). Because the trial-level domains (randomization process, deviations from intended interventions, and missing outcome data) were common to both outcomes, and the outcome-specific judgments for outcome measurement and selection of the reported result did not differ between PPCs and LOS in any included trial, a single set of judgments per study is presented in Figure 1. Outcomes synthesized narratively were not formally assessed with RoB 2.

**Figure 1:**
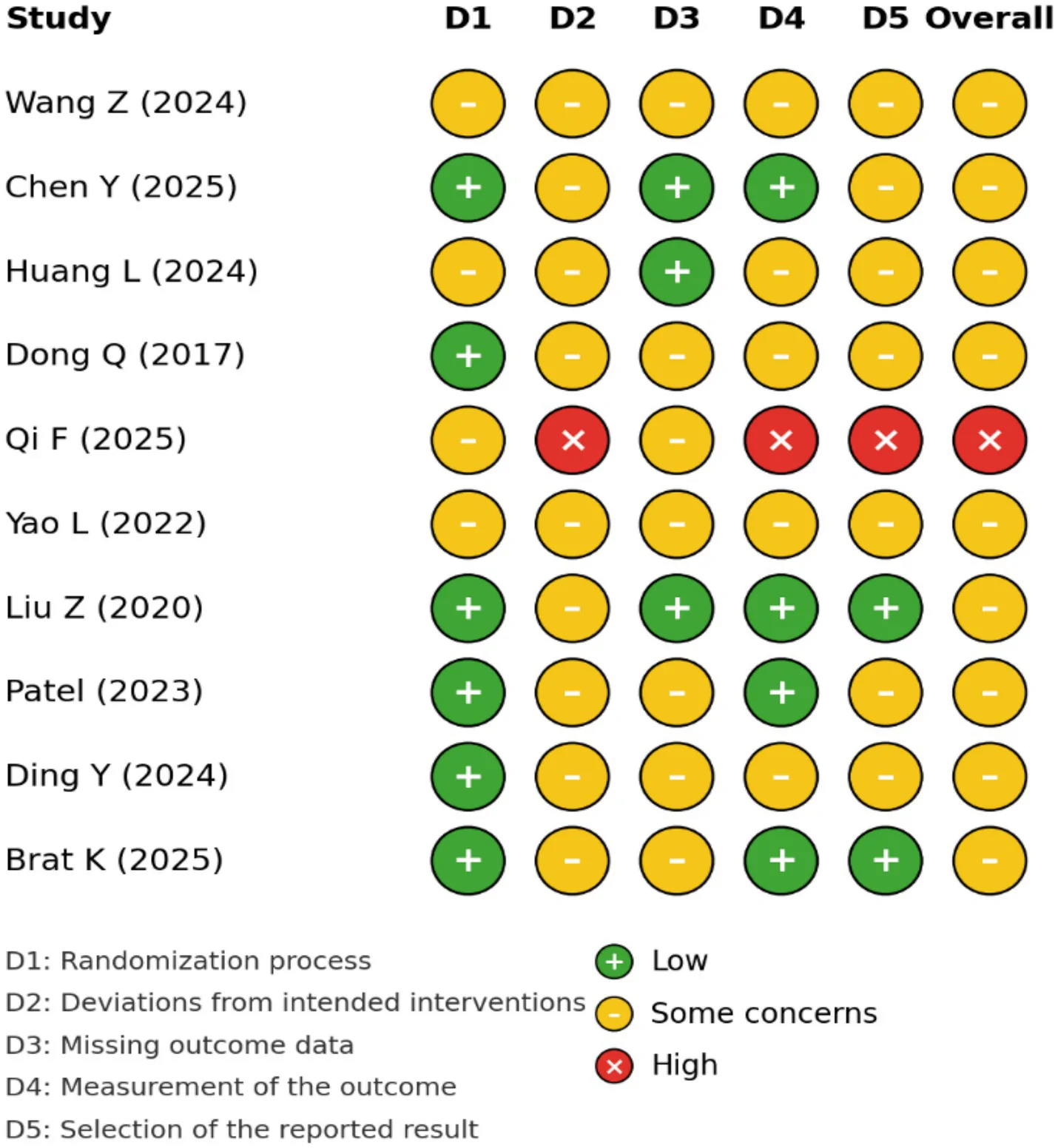
Flow diagram of the study. Adapted from the PRISMA 2020 flow diagram (37).

Five methodological domains were evaluated: bias arising from the randomization process; bias due to deviations from intended interventions; bias due to missing outcome data; bias in measurement of the outcome; bias in selection of the reported result.

Each domain was judged as low risk of bias, some concerns, or high risk of bias according to the RoB 2 guidance. Disagreements were resolved through consensus. Risk-of-bias figures were generated using the robvis visualization tool.

The certainty of evidence for each outcome was evaluated using the Grading of Recommendations Assessment, Development and Evaluation (GRADE) approach, and Summary of Findings tables were generated using GRADEpro GDT.

### Statistical analysis

Meta-analyses were performed using the meta suite in StataNow 19 BE.

Random-effects models with restricted maximum likelihood (REML) estimation were used to calculate pooled effect sizes. For dichotomous outcomes (PPCs), pooled odds ratios (ORs) with 95% confidence intervals (CIs) were calculated. Continuous outcomes (LOS) were synthesized using mean differences (MDs) with corresponding 95% CIs.

Statistical heterogeneity was assessed using Cochran’s Q test, I^2^ statistics, τ^2^, and 95% prediction intervals (PIs). I^2^ values of approximately 0–40%, 30–60%, 50–90%, and 75–100% were considered to represent potentially unimportant, moderate, substantial, and considerable heterogeneity, respectively, while interpretation also considered the magnitude and direction of effects and the strength of evidence for heterogeneity. When substantial heterogeneity was identified, potential sources were explored using prespecified subgroup and sensitivity analyses, where feasible. Prediction intervals were additionally examined to assess the expected range of treatment effects across different clinical settings. Although the prespecified protocol indicated narrative synthesis for outcomes with I^2^ > 75%, a random-effects meta-analysis of LOS was additionally performed as an exploratory analysis to summarize the direction and magnitude of the observed effects. This constituted a deviation from the prespecified protocol. Accordingly, the pooled LOS estimate was interpreted cautiously, with particular consideration of the magnitude of heterogeneity and the 95% PI, rather than relying solely on the statistical significance of the pooled estimate.

Considering the relatively small number of included studies, additional sensitivity analyses were conducted using the Hartung–Knapp–Sidik–Jonkman (HKSJ) adjustment.

Prespecified subgroup analyses were conducted according to: intervention type (ERAS vs. prehabilitation); intervention provider (multidisciplinary team vs. nurse-led); study region; risk of bias; definition of LOS (postoperative LOS vs. total hospital LOS).

Subgroup analyses according to surgical approach, number of ERAS components, and publication year were not performed because of insufficient reporting across studies. Meta-regression was not conducted because fewer than 10 studies were available for each outcome.

Sensitivity analyses included comparisons with fixed-effect models, exclusion of studies at high risk of bias, and leave-one-out analyses.

Publication bias was evaluated using contour-enhanced funnel plots. Harbord’s regression test was applied for PPCs, whereas Egger’s regression test was used for LOS.

Because an insufficient number of studies reported patient-reported or functional recovery outcomes using comparable instruments, these outcomes were synthesized narratively according to the Synthesis Without Meta-analysis (SWiM) reporting guideline (22). Patient-reported outcomes (anxiety, depression, postoperative pain, and quality of life) were distinguished from clinician-measured functional recovery outcomes (pulmonary function [FEV₁ and FVC] and time to first ambulation).

### Ethical considerations

As this study synthesized data from previously published studies and involved no direct participant recruitment or identifiable personal information, institutional review board approval was waived. Ethical exemption was granted by the Institutional Review Board of the authors’ institution (IRB No. 1041078-20260324-HR-119).

## Results

### Study selection

The literature search identified 1,852 records. After removing 588 duplicate records (532 using EndNote and 56 using Rayyan), 1,264 records underwent title and abstract screening. Of these, 1,217 records were excluded. Of the 1,217 records excluded during title and abstract screening, 253 were trial registration or protocol records without published results that had been retrieved through the bibliographic databases, chiefly CENTRAL, which indexes ClinicalTrials.gov and WHO ICTRP records. In addition, direct searches of ClinicalTrials.gov (*n* = 33) and the WHO ICTRP (*n* = 21) identified 52 unique registered trials, none of which had published results meeting the inclusion criteria.

A total of 47 articles were considered eligible for full-text assessment. Three articles for which full texts could not be obtained were excluded, leaving 44 studies for full-text review.

Following full-text assessment, studies were excluded because they evaluated single-component interventions (*n* = 25), used active comparator interventions (*n* = 3), were study protocols (*n* = 3), observational studies (*n* = 3), or were published in languages other than English or Korean (Chinese; *n* = 2). Backward citation searching of prior reviews and included studies identified five additional reports; three were excluded (one Chinese-language publication, one published before January 2015, and one with an ineligible population), and two randomized controlled trials were included. Consequently, 10 randomized controlled trials (RCTs) were included in the final systematic review and meta-analysis (Figure 2).

**Figure 2:**
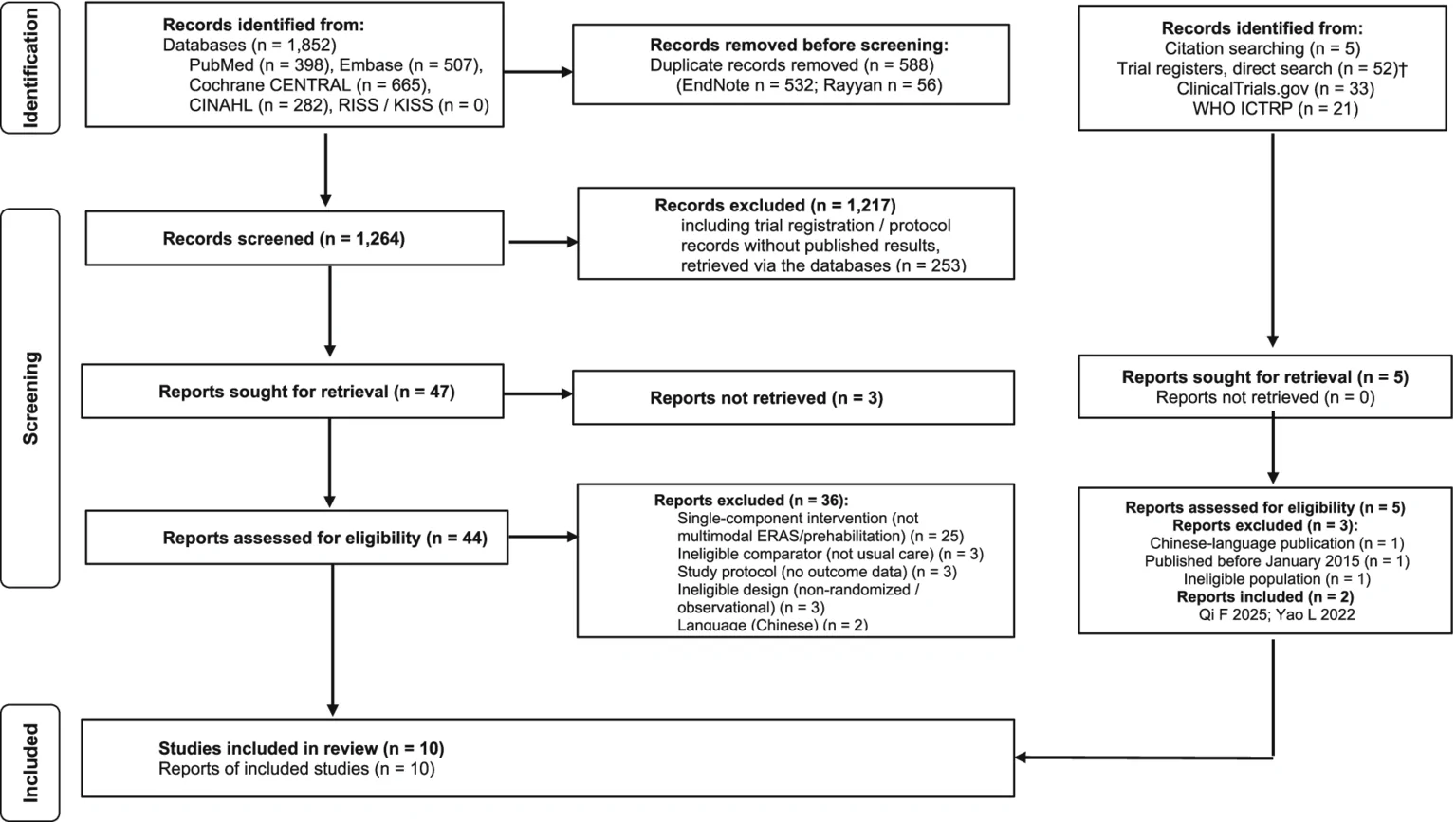
PRISMA 2020 flow diagram of the study selection process.

### Study characteristics

The characteristics of the included studies are summarized in Table 1.

**Table 1:** General characteristics of the included studies.

Study	Country	Design/setting	Trial reg.	Recruitment	N randomized (analyzed) I/C	Population (cancer; approach)	Funding/COI
Dong et al. (23)	China (Harbin)	Single-center RCT (block)	NR	2012.06–2014.03	35 (17/18)	NSCLC, ASA I–II; open pneumonectomy	Self-funded; COI Y
Wang et al. (24)	China (Wuhu)	Single-center RCT	NR (ethics #)	2020.01–2021.12	200 (100/100)	≥60 y NSCLC; thoracoscopic radical	NR; COI NR
Huang et al. (25)	China (Jinhua)	Single-center RCT	NR	2019.06–2022.12	85 (43/42)	NSCLC TNM I–II, <5 cm; thoracoscopic	None declared; COI Y
Ding et al. (26)	China (Jinan)	Single-center RCT	NR	2020.07–2022.06	611 (305/306)	Lung lesions (87% malignant); VATS	NR
Chen et al. (27)	China (Chongqing)	Single-center RCT (double-blind)	ChiCTR2400084551	2021.08–2022.07	80 (40/40)	Stage I–II NSCLC, 18–70 y; lobectomy	Grants; COI Y
Qi et al. (28)	China (Tangshan)	Single-center RCT	NR	2022.01–2024.01	120 (60/60)	Lung cancer; radical surgery	Provincial grant; COI Y
Liu et al. (29)	China (Beijing)	Single-center RCT	NCT03068507	2017.03–2017.12	73 (37/36)	Lung cancer; VATS	PUMC; COI Y
Yao et al. (30)	China (Shanghai)	Single-center RCT	NR	2021.06–2021.12	148 (74/74)	NSCLC I–II; VATS lobectomy	NR
Patel et al. (31)	Canada (Hamilton)	Single-center RCT (early stop)	NCT03689634	2018.10–2020.11	102 (51/51); analyzed 95 (45/50)	Lung cancer resection candidates	Grant; COI Y
Brat et al. (32)	Czechia + USA	Multi-center RCT	NCT04826575	2021.06–2024.06	132 (randomized); analyzed 122 (58/64)	Planned lung resection, high-risk	Institutional; COI Y

Study	Intervention & timing	ERAS elements (Pre/Intra/Post; Σ)	Provider	Comparator	PPC events I/C (OR, 95% CI)	LOS MD (days; definition)
Dong et al. (23)	Perioperative ERAS (FTS)	3/3/4 (Σ10)	Surgeon	Usual care	6/17 vs. 4/18 (1.91, 0.43–8.48)	−9.30 (total)
Wang et al. (24)	Perioperative ERAS, nurse-led	3/4/3 (Σ10)	Nurse-led	Usual care	8/100 vs. 18/100 (0.40, 0.16–0.96)	NR
Huang et al. (25)	Perioperative ERAS + nutrition	6/3/4 (Σ13)	Surgeon / Nutr.	Usual care	3/43 vs. 5/42 (0.55, 0.12–2.49)	−1.40 (postoperative)
Ding et al. (26)	Perioperative ERAS, MDT	4/1/5 (Σ10)	MDT	Usual care	35/305 vs. 70/306 (0.44, 0.28–0.68)	−4.00 (postoperative)
Chen et al. (27)	Perioperative ERAS, MDT	5/6/6 (Σ17)	MDT	Usual care	1/40 vs. 7/40 (0.12, 0.01–1.03)	−4.20 (postoperative)
Qi et al. (28)	Perioperative ERAS (high RoB)	2/2/4 (Σ8)	Surgeon	Usual care	13/60 vs. 16/60 (0.76, 0.33–1.76)	−0.42 (total)
Liu et al. (29)	Prehabilitation (4-domain)	3/0/0 (Σ3)	MDT	Usual care	4/37 vs. 5/36 (0.75, 0.18–3.06)	0.00 (postoperative)
Yao et al. (30)	Prehabilitation (trimodal)	3/0/0 (Σ3)	Nurse-led	Usual care	2/74 vs. 8/74 (0.23, 0.05–1.12)	−2.41 (postoperative)
Patel et al. (31)	Prehabilitation (multimodal)	4/0/0 (Σ4)	MDT	Usual care	NR (composite only)	−1.77 (postoperative)
Brat et al. (32)	Prehabilitation (multimodal)	3/0/0 (Σ3)	MDT	Usual care	20/58 vs. 35/64 (0.44, 0.21–0.91)	−1.67 (total)

NSCLC, non-small cell lung cancer; ASA, American Society of Anesthesiologists; VATS, video-assisted thoracoscopic surgery; COI, conflict of interest; NR, not reported; PUMC, Peking Union Medical College.

A total of 1,586 participants were randomized and 1,569 were analyzed across the 10 trials. Patel et al. (31) was terminated early because of COVID-19 (102 randomized, 95 analyzed), and Brat K 2025 randomized 132 participants of whom 122 completed the study.

ERAS, enhanced recovery after surgery; FTS, fast-track surgery; MDT, multidisciplinary team; PPC, postoperative pulmonary complication; OR, odds ratio; CI, confidence interval; LOS, length of hospital stay; MD, mean difference; RoB, risk of bias; NR, not reported.

Odds ratios are the uncorrected estimates entered into the random-effects meta-analysis and correspond exactly to the study-level estimates displayed in Figure 3. LOS definitions correspond to the subgroup stratification shown in Figure 4. Chen Y 2025 reported both total length of stay (8.65 ± 2.032 vs 18.20 ± 4.592 days; MD −9.55) and postoperative length of stay (5.45 ± 1.518 vs 9.65 ± 2.833 days; MD −4.20); the postoperative value was used in the primary analysis. Patel et al. (31) reported composite adverse events only and could not be included in the PPC meta-analysis.

The 10 RCTs were published between 2017 and 2025. Eight studies were conducted in China, whereas one study each was conducted in Canada and the Czech Republic (23–32).

Six studies evaluated perioperative ERAS protocols (23–28), and four investigated prehabilitation programs (29–32). Eight interventions were delivered by multidisciplinary teams (23, 25–29, 31, 32), whereas two adopted nurse-led models (24, 30).

Sample sizes ranged from 35 to 611 participants. All studies enrolled patients undergoing pulmonary resection for known or suspected lung cancer and compared ERAS-based or prehabilitation interventions with conventional perioperative care. Eight trials restricted enrolment to patients with primary lung cancer; Ding Y 2024 enrolled a broader lung-resection population in which malignant disease accounted for 87% of participants, satisfying the prespecified threshold of ≥80%, and Brat K 2025 enrolled patients scheduled for lung resection because of confirmed or highly suspected lung malignancy.

### Risk of bias

Nine studies were judged as having some concerns, primarily because of potential bias arising from deviations from intended interventions and outcome measurement. One study (28) was judged to have a high risk of bias owing to baseline imbalance, lack of blinding, absence of prospective registration, and concerns regarding outcome reporting.

Sensitivity analyses excluding this study were performed. Detailed domain-level assessments are presented in Figure 1.

### Effects of the interventions

#### Postoperative pulmonary complications

Nine studies involving 1,474 participants reported PPCs.

The pooled analysis demonstrated that ERAS and prehabilitation significantly reduced the risk of PPCs compared with conventional care (OR = 0.48, 95% CI 0.36–0.65, *p* < 0.001) (Figure 3).

**Figure 3:**
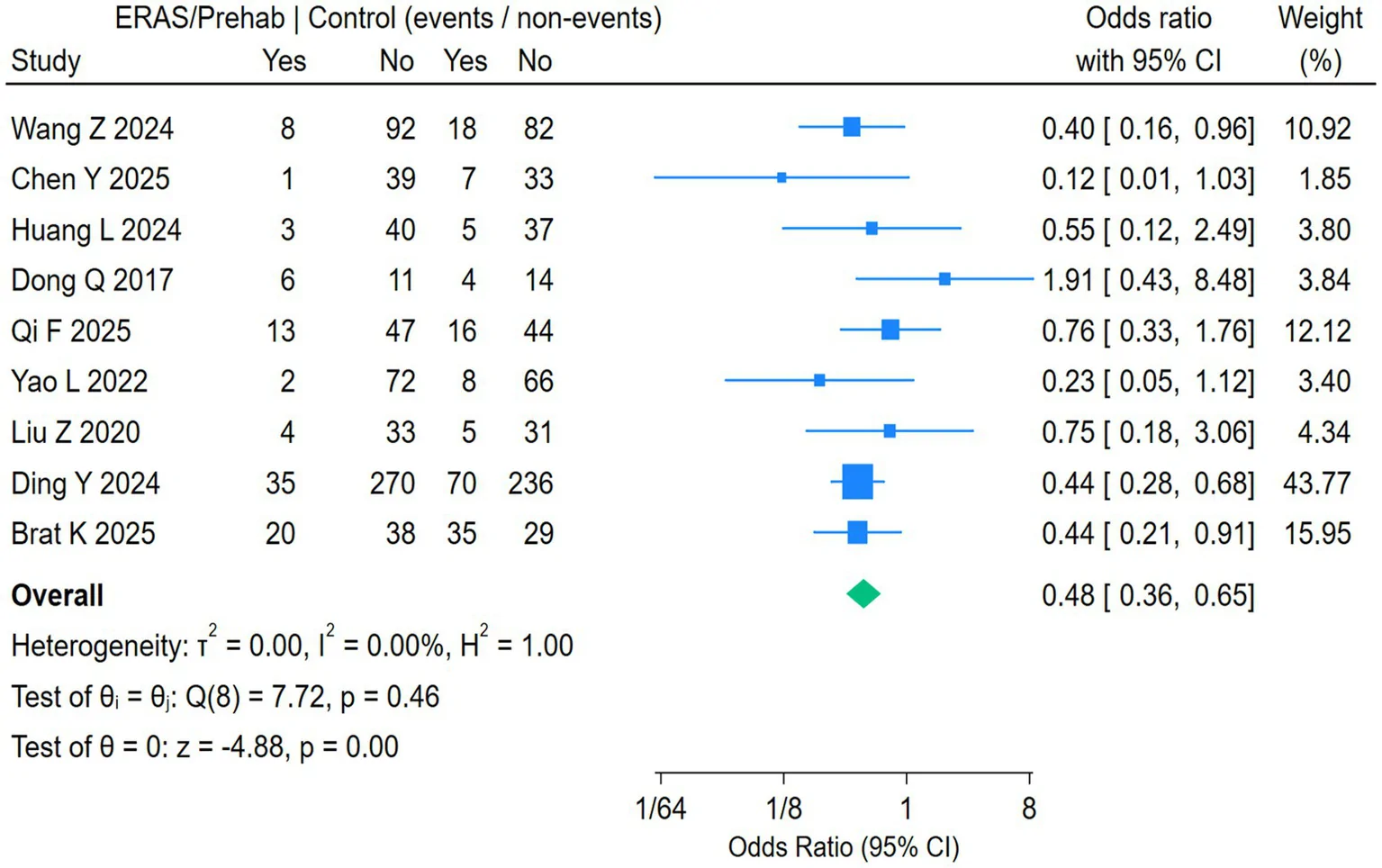
Forest plot of the effect of the intervention on postoperative pulmonary complications (random-effects model, REML).

No statistical heterogeneity was observed (*I*^2^ = 0.0%, τ^2^ = 0.00, Q = 7.72, *p* = 0.460). Similar findings were obtained using the fixed-effect model (OR = 0.48, 95% CI 0.36–0.63).

Leave-one-out analyses yielded pooled effect estimates ranging from OR 0.45 to 0.52, indicating that no individual study substantially influenced the overall results. Excluding the study at high risk of bias also produced comparable findings.

Prespecified subgroup analyses according to intervention type showed that the pooled OR was 0.50 (95% CI 0.36–0.69) for ERAS and 0.44 (95% CI 0.24–0.80) for prehabilitation. No statistically significant subgroup difference was detected (p for subgroup difference = 0.720). Given the small number of studies within each subgroup, this finding should not be interpreted as evidence of equivalent effects between ERAS and prehabilitation.

#### Length of hospital stay

Nine studies reported hospital length of stay. Because the definition of LOS differed across studies, six studies reporting postoperative LOS (*n* = 1,092) were included in the primary analysis, whereas three studies reporting total hospital LOS were analyzed separately in subgroup analyses (Figure 4).

**Figure 4:**
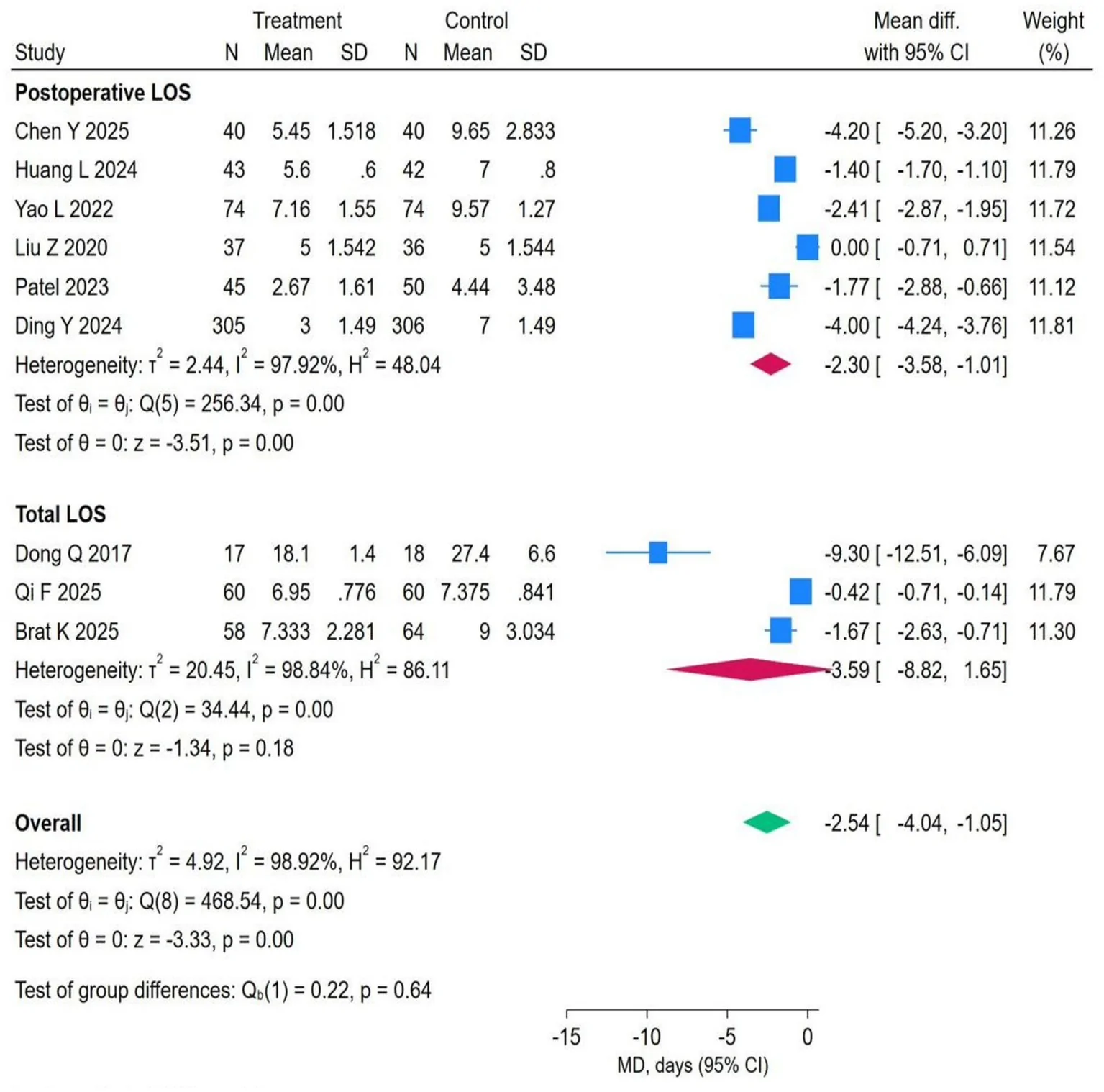
Forest plot of length of hospital stay analyzed as a continuous outcome using mean differences (MDs, days) with 95% confidence intervals, stratified by the definition of LOS (postoperative LOS vs. total hospital LOS). The vertical line at MD = 0 represents no difference between groups.

As this quantitative synthesis represented a deviation from the prespecified protocol, the pooled LOS estimate was considered exploratory. The random-effects analysis yielded a pooled MD of −2.30 days (95% CI − 3.58 to −1.01); however, heterogeneity was considerable (I^2^ = 97.9%), and the 95% PI (−7.00 to 2.41 days) crossed the null, indicating substantial variability in the expected effect across clinical settings.

Sensitivity analyses produced consistent findings across analytical approaches, including fixed-effect models (MD = −2.78 days), DerSimonian–Laird estimation (MD = −2.30 days), and leave-one-out analyses (MD ranging from −1.93 to −2.75 days). Egger’s regression test did not indicate evidence of publication bias (z = 0.11, *p* = 0.91). Subgroup analysis showed a greater reduction in LOS among ERAS studies (MD = −3.17 days, 95% CI − 4.96 to −1.38) than among prehabilitation studies (MD = −1.40 days, 95% CI − 2.86 to 0.06); however, the subgroup difference was not statistically significant (*p* = 0.13). Likewise, no significant subgroup difference was observed according to the definition of LOS (*p* = 0.64).

#### Sensitivity analyses

The principal findings remained robust across all sensitivity analyses (Table 2).

**Table 2:** Sensitivity analyses.

Outcome	Analysis	Result
PPCs	Fixed effect (Mantel–Haenszel)	OR 0.48 (0.36, 0.63) — consistent with the random-effects estimate
	Leave-one-out	Pooled OR 0.45 to 0.52; all 95% CIs excluded 1.0 (direction consistent)
	High RoB excluded (Qi F, 2025)	OR 0.45 (0.33, 0.62), 8 studies, *I*^2^ = 0% — effect maintained (Supplementary Figure S4)
	Intervention-timing subgroup	Perioperative ERAS OR 0.50 (0.36, 0.69) vs. prehabilitation OR 0.44 (0.24, 0.80); Q_b_*p* = 0.72 — consistent, no between-group difference
	Small-study effects (Harbord)	*z* = 0.52, *p* = 0.61 — no evidence
LOS	Fixed effect / DerSimonian–Laird	MD − 2.78 / −2.30 (−3.62, −0.97) — point estimate stable
	95% prediction interval	−7.00 to +2.41 (includes zero) — wide between-study variance
	Leave-one-out	Pooled MD − 1.93 to −2.75; all 95% CIs excluded 0 (direction consistent)
	High RoB excluded (all 8 LOS studies)	MD − 2.83 (−4.45, −1.21) — direction maintained (Supplementary Figure S7)
	Studies using Wan-converted values excluded (4 studies)	MD − 2.41 (−3.59, −1.23) — direction maintained (Supplementary Figure S6)
	Intervention-timing subgroup	Perioperative ERAS −3.17 (−4.96, −1.38) vs. prehabilitation −1.40 (−2.86, +0.06); Q_b_*p* = 0.13
	Definition-based subgroup	Postoperative vs. total: Q_b_*p* = 0.64 (definition did not explain heterogeneity)
	Small-study effects (Egger)	*z* = 0.11, *p* = 0.91 — no evidence
HKSJ correction (pre-specified)	CI adjustment (REML)	PPC OR 0.48 (0.34, 0.68); LOS MD − 2.30 (−3.98, −0.61) — neither crossed zero

PPCs, Postoperative pulmonary complications; Los, Length of hospital stay; MD, mean difference; OR, odds ratio; Q_b_, between-group heterogeneity statistic; REML, restricted maximum likelihood. Funnel-plot–based tests (Harbord, Egger) are exploratory because fewer than 10 studies were available per outcome.

After excluding the study judged to be at high risk of bias, the pooled estimate for PPCs remained significant (OR = 0.45, 95% CI 0.33–0.62; I^2^ = 0%). Similar results were obtained for LOS. Excluding studies requiring statistical conversion of summary data also did not materially alter the pooled estimates.

Application of the Hartung–Knapp–Sidik–Jonkman (HKSJ) adjustment yielded findings consistent with the primary analyses for both PPCs (OR = 0.48, 95% CI 0.34–0.68) and LOS (MD = −2.30 days, 95% CI − 3.98 to −0.61).

### Certainty of evidence

According to the GRADE assessment, the certainty of evidence was moderate for postoperative pulmonary complications, downgraded one level for risk of bias because most trials were judged to have some concerns, and low for length of hospital stay, downgraded for risk of bias and for inconsistency reflecting the substantial statistical heterogeneity. Certainty was not formally graded for outcomes synthesized narratively.

### Narrative synthesis of patient-reported and functional recovery outcomes

Because of substantial heterogeneity in outcome measures and assessment time points, patient-reported and functional recovery outcomes were synthesized narratively (Table 3). Patient-reported outcomes comprised anxiety, depression, postoperative pain, and quality of life, whereas pulmonary function (FEV₁ and FVC) and time to first ambulation were classified as clinician-measured functional recovery outcomes. The direction of effects generally favored ERAS or prehabilitation across these outcomes; where numeric estimates were reported by the individual trials, they are summarized in Table 3. Quantitative pooling was not feasible because of heterogeneity in measurement instruments and assessment schedules, and the synthesis therefore primarily reflects the direction rather than the magnitude of effects. Overall, the direction of effects generally favored ERAS or prehabilitation across the reported patient-reported and functional recovery outcomes, although differences in measurement instruments and assessment time points precluded quantitative comparison of effect magnitude.

**Table 3:** Patient-reported and functional recovery outcomes.

Outcome	Measurement instrument	Direction of effect (narrative synthesis)
Anxiety	SAS, HADS-A, STAI	Tendency toward reduction in the intervention group
Depression	SDS, HADS-D	Tendency toward reduction in the intervention group
Postoperative pain	VAS, NRS	Tendency toward relief in the intervention group (MD approximately −0.5 to −1.6 in studies reporting numeric values)
Quality of life	SF-36, EORTC QLQ-C30, FACT-L	Tendency toward improvement in the intervention group
Pulmonary function	FEV1, FVC	Tendency toward improvement in the intervention group
Time to first ambulation	Timing of postoperative ambulation	Tendency toward shortening (earlier return to activity) in the intervention group

Because of heterogeneity in measurement instruments and assessment time points, quantitative pooling was not performed; the direction of effect represents a narrative synthesis conducted according to the SWiM guideline. HADS, hospital anxiety and depression scale; SAS, self-rating anxiety scale; STAI, state–trait anxiety inventory; SDS, self-rating depression scale; VAS, visual analog scale; NRS, numeric rating scale; SF-36, 36-item short-form survey; EORTC QLQ-C30, European Organization for Research and Treatment of Cancer Quality of Life Questionnaire; FACT-L, functional assessment of cancer therapy–lung; FEV1, forced expiratory volume in 1 s; FVC, forced vital capacity. Anxiety, depression, postoperative pain, and quality of life are patient-reported outcomes; pulmonary function (FEV₁, FVC) and time to first ambulation are clinician-measured functional recovery outcomes.

## Discussion

This systematic review and meta-analysis synthesized evidence from 10 randomized controlled trials involving 1,569 patients undergoing pulmonary resection for lung cancer to evaluate the effectiveness of ERAS and prehabilitation (23–32). The findings demonstrated that ERAS and prehabilitation were associated with a significantly lower risk of PPCs compared with conventional perioperative care. The exploratory pooled analysis also suggested a shorter LOS; however, this finding was characterized by considerable between-study heterogeneity and a 95% prediction interval crossing the null and should therefore be interpreted cautiously.

The prevention of PPCs represents one of the primary goals of perioperative management in thoracic surgery because pulmonary complications are strongly associated with prolonged hospitalization, increased healthcare costs, and postoperative mortality (5–7). In the present study, ERAS and prehabilitation reduced the risk of PPCs by approximately 52%, indicating a clinically meaningful benefit. Although these interventions are implemented at different stages of the surgical pathway, they appear to improve postoperative recovery through complementary mechanisms. ERAS minimizes the physiological stress response by integrating evidence-based perioperative care elements such as multimodal analgesia, early mobilization, respiratory exercises, optimized fluid management, and early oral nutrition (13–15). In contrast, prehabilitation enhances patients’ physiological reserve before surgery through exercise training, inspiratory muscle training, nutritional optimization, and psychological support. Together, these approaches improve patients’ resilience to surgical stress, thereby reducing the likelihood of postoperative respiratory complications (16–18).

An important finding of this review is the remarkable consistency of the effect on PPCs. No statistical heterogeneity was observed, despite variations in healthcare systems, intervention protocols, and study settings. Furthermore, sensitivity analyses, including leave-one-out analyses and exclusion of the study judged to be at high risk of bias, yielded nearly identical effect estimates. These findings strengthen confidence in the robustness and reproducibility of the observed treatment effect and suggest that the beneficial impact of structured perioperative care extends across diverse clinical settings (33).

The exploratory pooled analysis suggested an average reduction in postoperative LOS of 2.3 days. However, this finding should be interpreted with considerable caution because heterogeneity was extremely high (I^2^ = 97.9%) and the 95% PI crossed the null. Nevertheless, earlier discharge may facilitate faster return to daily activities, reduce the risk of hospital-acquired complications, improve bed availability, and decrease healthcare expenditures (34). Given the increasing incidence of lung cancer and the growing demand for thoracic surgical services in aging populations, these findings support the adoption of evidence-based perioperative pathways to improve healthcare efficiency (35).

Nevertheless, substantial statistical heterogeneity was observed for LOS. Considerable variation was also observed in the content and intensity of the ERAS and prehabilitation protocols across the included trials. The ERAS programs differed in the number and distribution of preoperative, intraoperative, and postoperative components, ranging from approximately 8 to 17 components, and included varying combinations of patient education, nutritional optimization, respiratory training, multimodal analgesia, fluid management, early feeding, mobilization, and chest tube management. The delivery models also differed, including surgeon-led, nurse-led, and multidisciplinary approaches. Similarly, prehabilitation programs varied in their duration and combination of exercise, inspiratory muscle training, nutritional support, and psychological interventions. These differences in intervention content, intensity, timing, and delivery may have contributed to between-study variability in postoperative outcomes, particularly LOS (36). Although no heterogeneity was observed for PPCs, the substantial heterogeneity in LOS suggests that the magnitude of benefit may depend partly on how ERAS or prehabilitation is operationalized within individual healthcare settings. Accordingly, the pooled LOS estimate should be interpreted as an average effect across heterogeneous multimodal protocols rather than as the effect of a single standardized intervention.

Subgroup analyses demonstrated that both perioperative ERAS pathways and prehabilitation programs significantly reduced PPCs, with no statistically significant difference between intervention types. Subgroup analyses did not identify statistically significant evidence of effect modification by intervention type. However, a non-significant test for subgroup differences does not demonstrate equivalence between ERAS and prehabilitation. This distinction is particularly important for LOS, for which the 95% CI for the prehabilitation subgroup included the null value. Moreover, the small number of studies within each subgroup limited the statistical power to detect potential subgroup differences. Accordingly, these subgroup findings should be considered exploratory and should not be interpreted as evidence of equivalent or comparable effectiveness between intervention types.

Although quantitative synthesis of patient-reported and functional outcomes was not feasible, the narrative synthesis consistently suggested favorable effects of ERAS and prehabilitation on anxiety, depression, postoperative pain, quality of life, pulmonary function, and functional recovery. Prehabilitation demonstrated relatively consistent benefits in cardiopulmonary function and postoperative functional recovery, whereas ERAS interventions were more frequently associated with improvements in psychological adaptation and overall postoperative recovery. These findings indicate that the benefits of these interventions extend beyond reducing postoperative complications to encompass broader aspects of recovery that are increasingly recognized as important PROs (36). However, substantial variability in measurement instruments and assessment time points limited direct comparisons between studies. Future research should incorporate standardized, validated PRO measures to facilitate quantitative synthesis.

This review has several important strengths. First, only randomized controlled trials were included, thereby minimizing the risk of confounding associated with observational studies. Second, methodological rigor was enhanced through the application of the Cochrane Risk of Bias 2 tool, GRADE methodology, prediction intervals, Hartung–Knapp–Sidik–Jonkman adjustment, and multiple sensitivity analyses. Third, unlike previous reviews that evaluated ERAS or prehabilitation separately, this review examined both strategies within a broader framework of multimodal perioperative optimization while preserving their distinction through intervention-specific subgroup analyses. This approach allowed the randomized evidence for both strategies relative to conventional perioperative care to be synthesized within a single review. However, because no head-to-head randomized comparisons were available, the findings should not be interpreted as evidence of the comparative effectiveness or equivalence of ERAS and prehabilitation.

Several limitations should also be acknowledged. Blinding of participants and healthcare providers was inherently difficult because of the nature of the interventions, introducing the possibility of performance and detection bias. In addition, most included studies were conducted in China, which may limit the generalizability of the findings to other healthcare systems. The substantial heterogeneity observed for LOS also reduced the certainty of evidence for this outcome. In addition, the search was restricted to studies published in English or Korean from January 2015 onward, as prespecified in the registered protocol; one pre-2015 multimodal fast-track randomized trial identified through citation searching was excluded on the basis of publication date, and Chinese-language reports were not eligible, which may have introduced language and time-period bias. Risk of bias was formally assessed for the two primary outcomes only, and the narrative synthesis of patient-reported and functional outcomes primarily reflects the direction rather than the magnitude of effects. Moreover, one trial enrolled a broader lung-resection population in which malignant disease accounted for 87% of participants, which may have slightly diluted lung cancer–specific effects. Finally, the relatively small number of included studies limited the ability to fully assess publication bias and to perform more detailed subgroup or meta-regression analyses.

Overall, the present evidence supports the implementation of ERAS and prehabilitation as effective perioperative strategies for patients undergoing pulmonary resection for lung cancer. Nevertheless, future multicenter randomized controlled trials involving diverse healthcare settings are warranted to establish the optimal components, timing, and intensity of these interventions and to develop standardized outcome measures, particularly for PROs and hospital LOS.

## Conclusion

This systematic review and meta-analysis of 10 randomized controlled trials demonstrated that both ERAS and prehabilitation significantly reduced postoperative pulmonary complications and shortened hospital length of stay among patients undergoing pulmonary resection for lung cancer. The beneficial effect on postoperative pulmonary complications was highly consistent across studies, whereas the reduction in length of stay should be interpreted with caution because of substantial between-study heterogeneity.

ERAS and prehabilitation should be considered distinct perioperative optimization strategies that may improve postoperative outcomes compared with conventional perioperative care. Although no statistically significant differences were detected between intervention-type subgroups, the present findings should not be interpreted as demonstrating equivalent effectiveness between ERAS and prehabilitation, because no direct head-to-head randomized comparisons were included and the number of studies within each subgroup was limited.

Future multicenter randomized controlled trials conducted in different healthcare systems are needed to establish the optimal components and timing of perioperative interventions, standardize outcome definitions, and strengthen the evidence regarding patient-reported outcomes and long-term postoperative recovery.

## Data Availability

The original contributions presented in the study are included in the article/supplementary material, further inquiries can be directed to the corresponding author.
