# Supplementary material for: Effects of enhanced recovery after surgery and prehabilitation interventions on postoperative outcomes after lung resection in patients with lung cancer: a systematic review and meta-analysis
Source: Front Public Health. 2026 Oct 5;14:1939340. doi: 10.3389/fpubh.2026.1939340 (PMC13677867; doi:10.3389/fpubh.2026.1939340)
Supplement: Supplementary file 1 [file Supplementary_file_1.pdf]

Supplementary material

Supplementary Table S1. GRADE Summary of Findings.

ERAS and prehabilitation compared with conventional perioperative care for adults undergoing pulmonary resection for lung cancer.

Outcome	No. of participants (studies)	Relative effect (95% CI)	Assumed risk - conventional care	Corresponding risk - ERAS/prehabilitation	Certainty (GRADE)	Comments
Postoperative pulmonary complications (PPCs)	1,474 (9 RCTs)	OR 0.48 (0.36 to 0.65)	227 per 1,000	124 per 1,000 (96 to 160); 103 fewer per 1,000 (from 131 fewer to 67 fewer)	⊕⊕⊕◯ MODERATE [a]	I^2 = 0%; consistent across all sensitivity and subgroup analyses
Length of hospital stay - postoperative (days)	1,092 (6 RCTs)	MD −2.30 (−3.58 to −1.01)	Mean postoperative stay 4.4–9.7 days across trials	On average 2.30 days shorter (from 3.58 to 1.01 days shorter)	⊕⊕◯◯ LOW [a,b]	I^2 = 97.9%; 95% prediction interval −7.00 to 2.41; direction consistent across all trials

GRADE Working Group grades of evidence: High — further research is very unlikely to change confidence in the estimate; Moderate — further research is likely to have an important impact; Low — further research is very likely to have an important impact. CI, confidence interval; OR, odds ratio; MD, mean difference; RCT, randomized controlled trial.

[a] Downgraded one level for risk of bias: all contributing trials were judged to have some concerns, and one trial was judged to be at high risk of bias; sensitivity analyses excluding the high risk-of-bias trial yielded consistent estimates.

[b] Downgraded one level for inconsistency: substantial statistical heterogeneity (I^2 = 97.9%) and a 95% prediction interval crossing the null (−7.00 to 2.41 days), attributable mainly to differences in intervention protocols, discharge practices, and healthcare systems.

The assumed risk is the pooled event rate in the conventional-care arms of the contributing trials (168/740). The corresponding risk (and its 95% CI) is based on the assumed risk and the relative effect of the intervention.

Supplementary Table S2. Characteristics of ongoing registered trials without available results.

Trial registration no.	Country / setting	Status at export	Target sample size	Intervention (brief)	Planned primary outcomes
NCT04451473	China (single centre)	Recruiting	350	Multimodal ERAS pathway vs. traditional perioperative management for lung surgery	ICU utilisation; pain; time to ambulation; time to oral feeding; length of hospitalisation; complications
NCT03271749	Brazil (Hospital Santa Marcelina, São Paulo)	Not yet recruiting	NR	PROSM multimodal accelerated-recovery pathway (preoperative, anaesthesia and postoperative care for lung resections) vs. traditional institutional care	Length of hospital stay; postoperative pain (VAS)
NCT04049942	China (Peking Union Medical College Hospital)	Recruiting (last known)	NR	Short home-based multimodal prehabilitation vs. preoperative aerobic training before VATS lobectomy (protocol published: Trials 2023; 10.1186/s13063-023-07220-4)	Perioperative 6-min walk distance

Trials were identified through direct searches of ClinicalTrials.gov (n = 33) and the WHO ICTRP (n = 21; 52 unique records) exported on 3 May 2026; the three trials listed here were relevant to the review question but had no published results available at the time of data extraction.

Supplementary Figure S1.

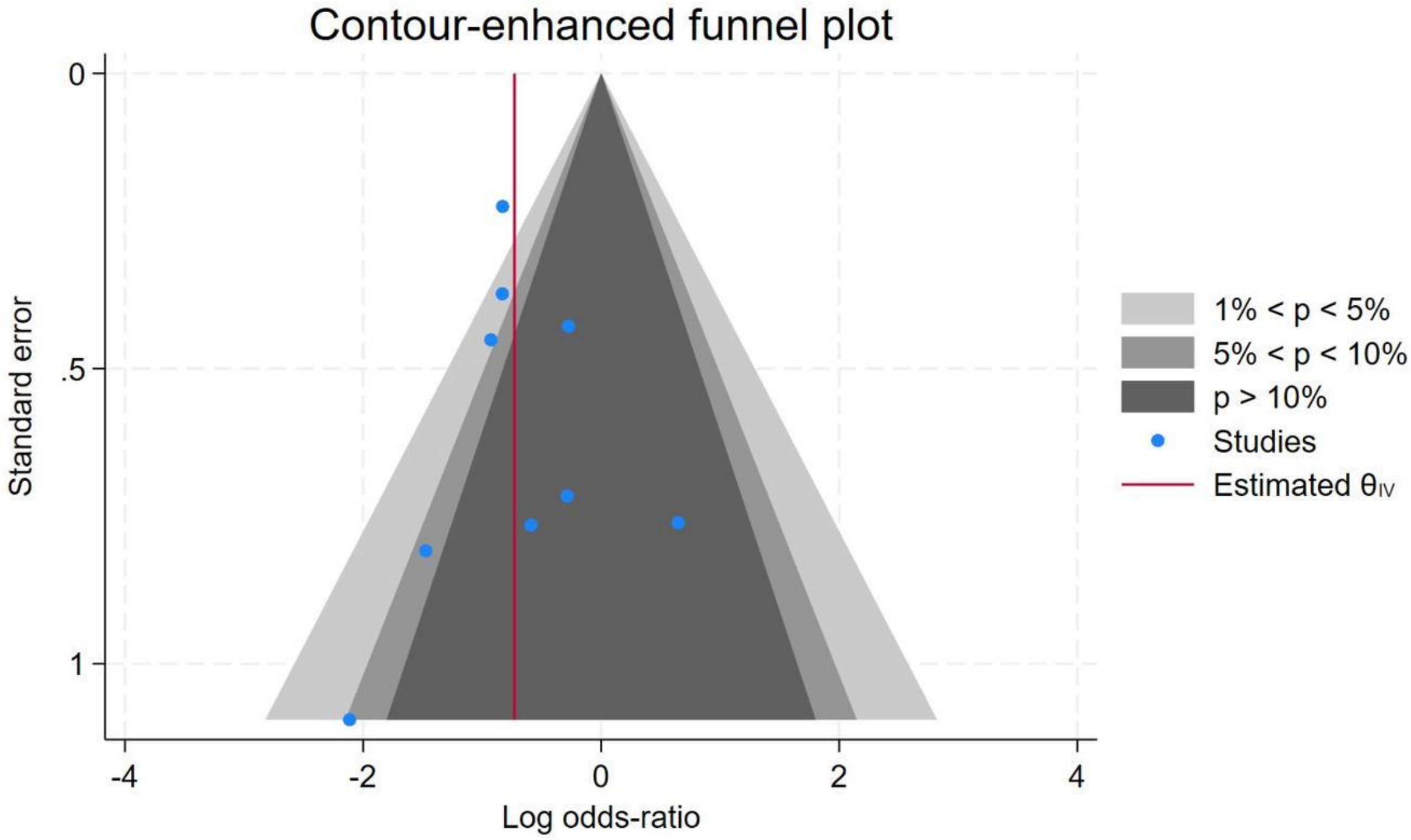

Contour-enhanced funnel plot for postoperative pulmonary complications (9 trials). Harbord's regression test showed no evidence of small-study effects ($z = 0.52$, $p = .61$).

Supplementary Figure S2.

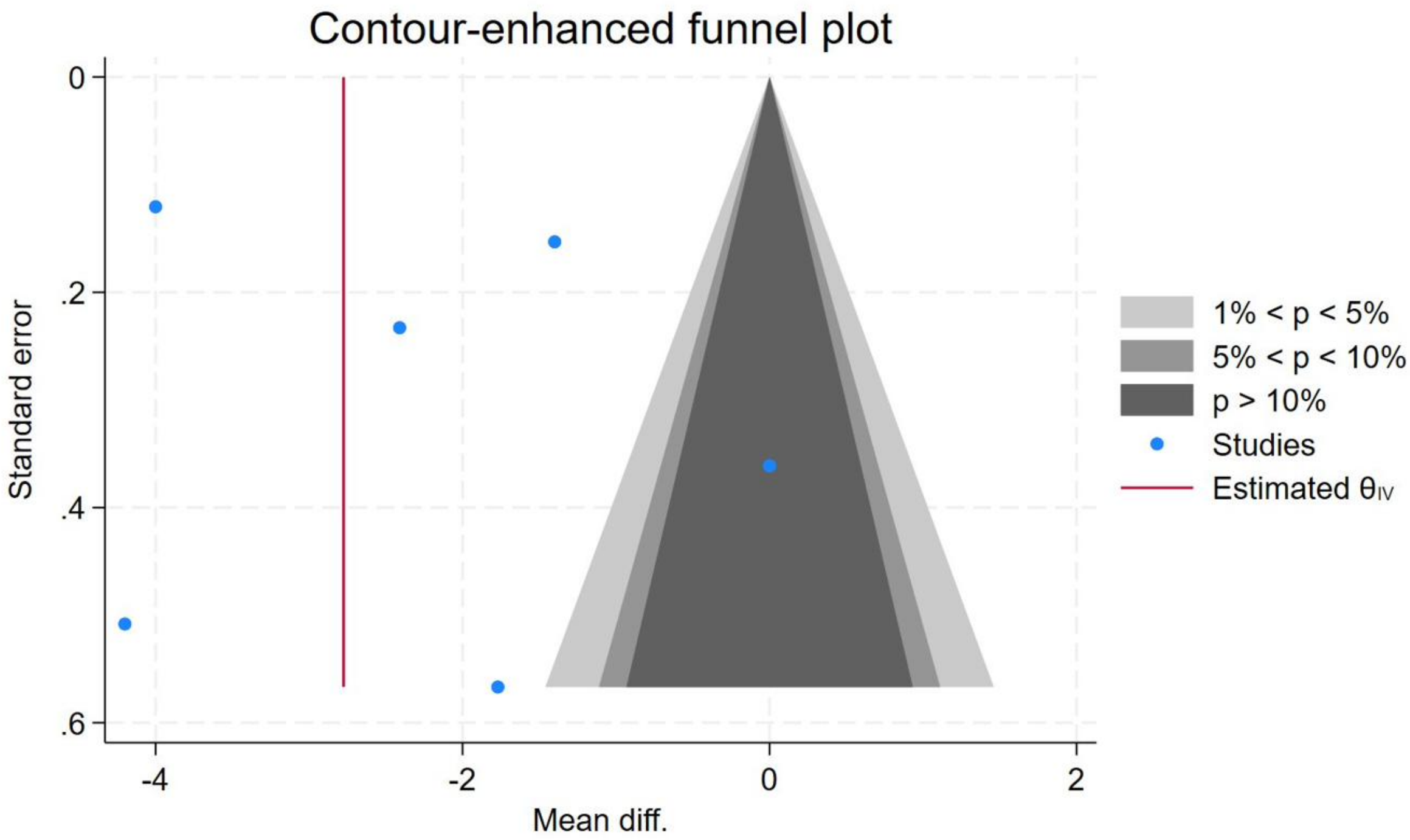

Contour-enhanced funnel plot for postoperative length of hospital stay (6 trials). Egger's regression test showed no evidence of small-study effects (z = 0.11, p = .91).

Supplementary Figure S3.

(A)

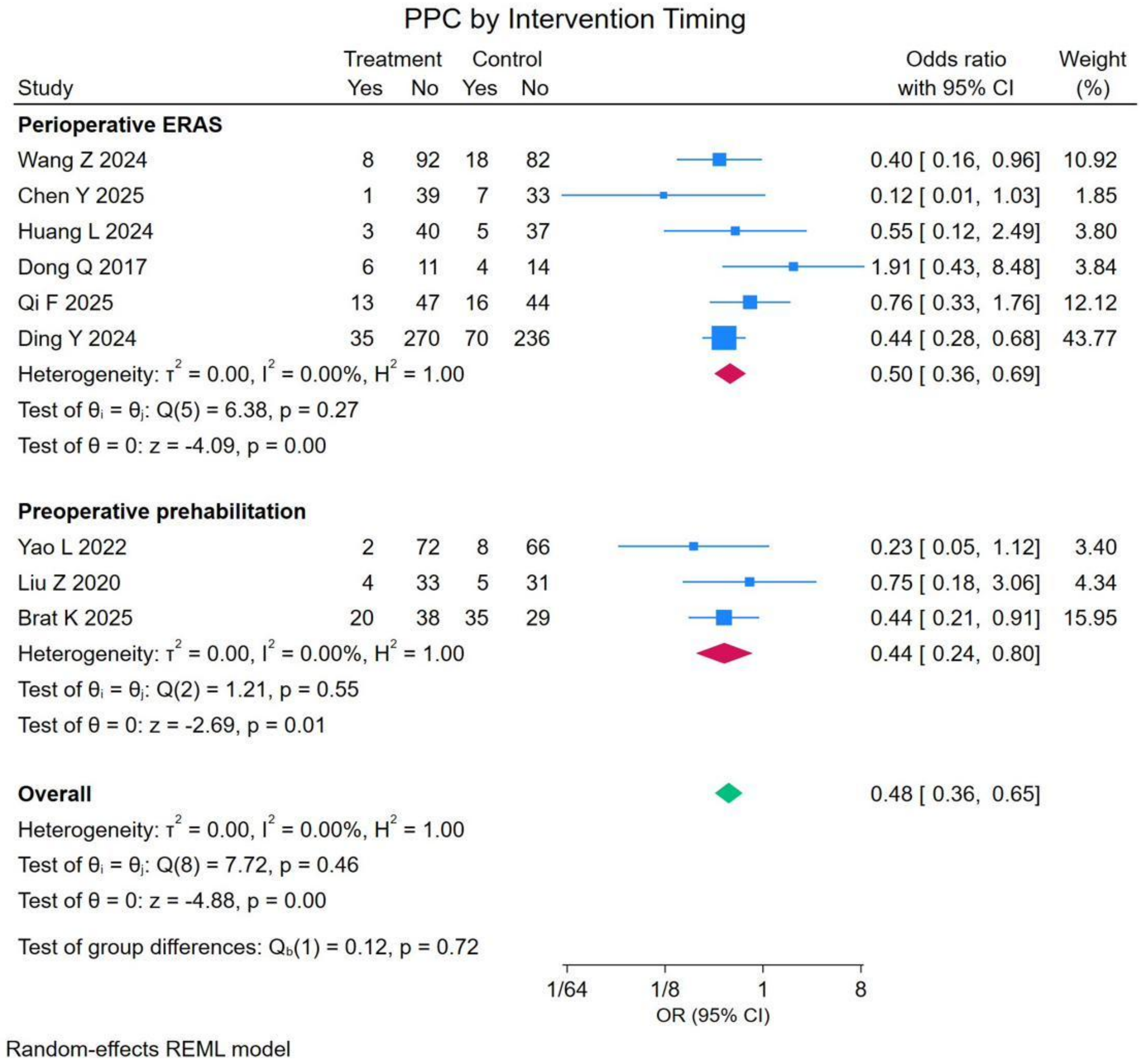

(B)

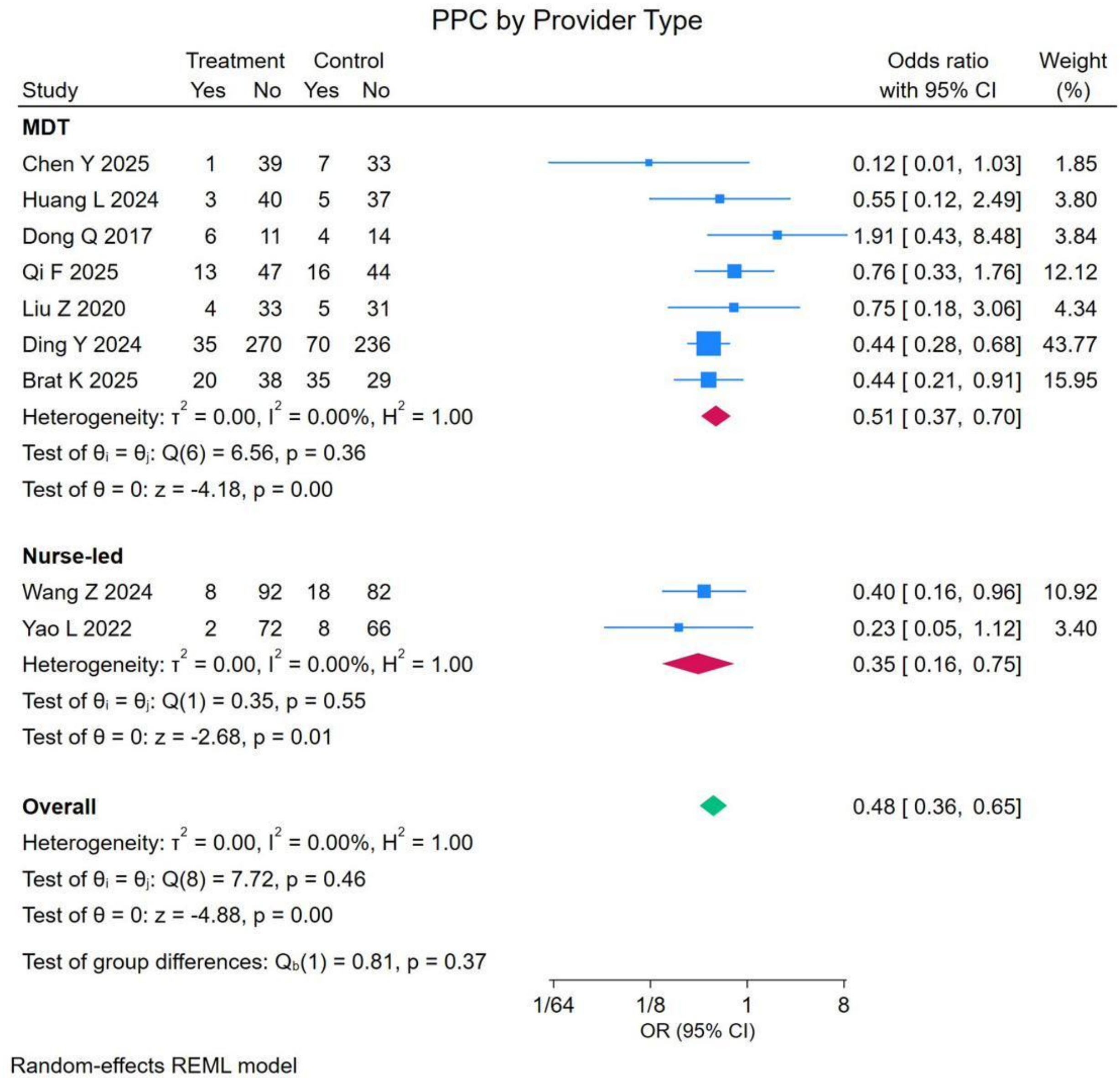

PPC by Provider Type
Study
Treatment Yes No
Control Yes No
Odds ratio with 95% CI
Weight (%)
MDT
Chen Y 2025 1 39 7 33 0.12 [0.01, 1.03] 1.85
Huang L 2024 3 40 5 37 0.55 [0.12, 2.49] 3.80
Dong Q 2017 6 11 4 14 1.91 [0.43, 8.48] 3.84
Qi F 2025 13 47 16 44 0.76 [0.33, 1.76] 12.12
Liu Z 2020 4 33 5 31 0.75 [0.18, 3.06] 4.34
Ding Y 2024 35 270 70 236 0.44 [0.28, 0.68] 43.77
Brat K 2025 20 38 35 29 0.44 [0.21, 0.91] 15.95
Heterogeneity: τ² = 0.00, I² = 0.00%, H² = 1.00 0.51 [0.37, 0.70]
Test of θi = θj: Q(6) = 6.56, p = 0.36
Test of θ = 0: z = -4.18, p = 0.00
Nurse-led
Wang Z 2024 8 92 18 82 0.40 [0.16, 0.96] 10.92
Yao L 2022 2 72 8 66 0.23 [0.05, 1.12] 3.40
Heterogeneity: τ² = 0.00, I² = 0.00%, H² = 1.00 0.35 [0.16, 0.75]
Test of θi = θj: Q(1) = 0.35, p = 0.55
Test of θ = 0: z = -2.68, p = 0.01
Overall 0.48 [0.36, 0.65]
Heterogeneity: τ² = 0.00, I² = 0.00%, H² = 1.00
Test of θi = θj: Q(8) = 7.72, p = 0.46
Test of θ = 0: z = -4.88, p = 0.00
Test of group differences: Qb(1) = 0.81, p = 0.37
1/64
1/8
1
8
OR (95% CI)
Random-effects REML model

(C)

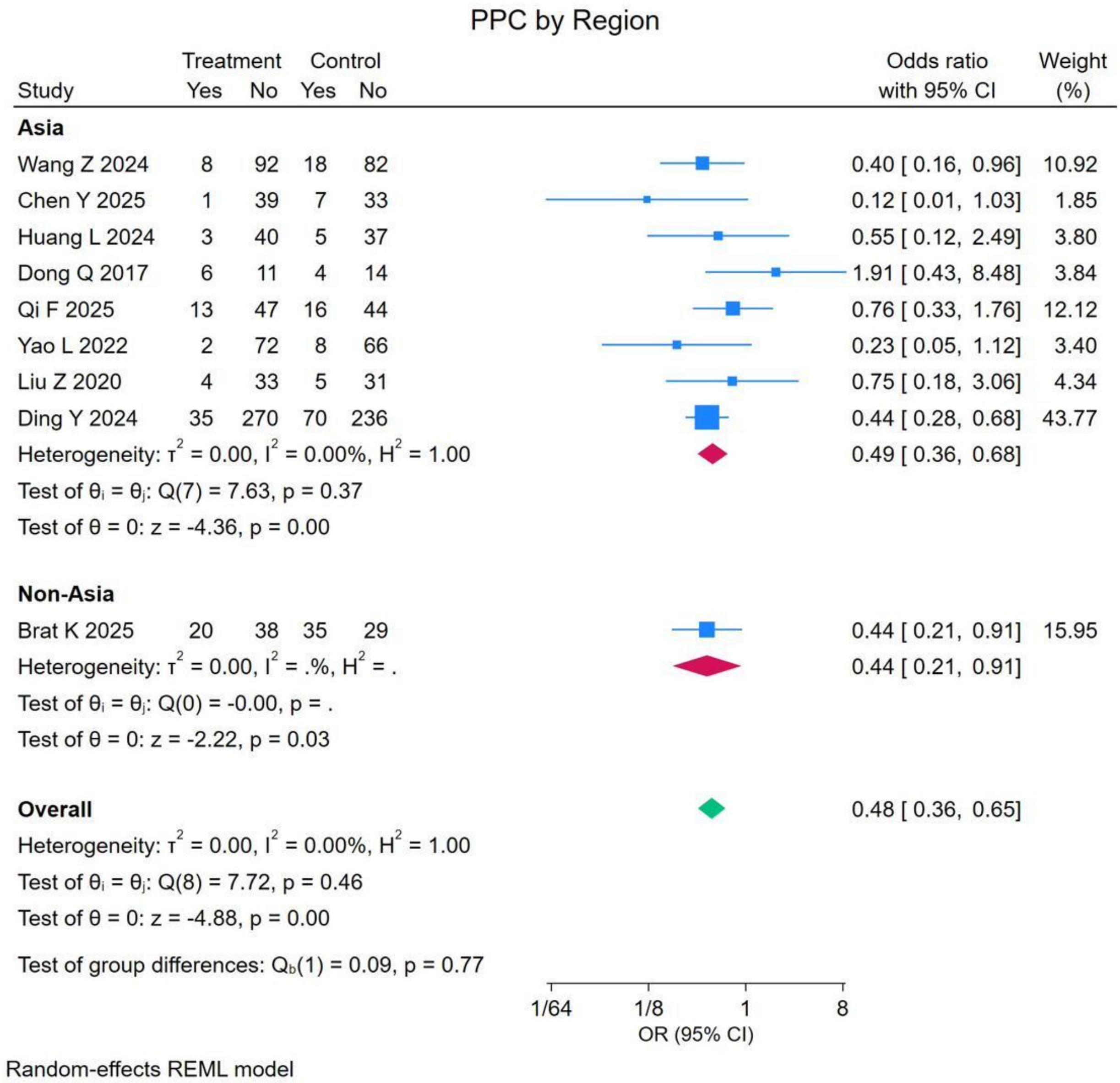

Subgroup analyses for postoperative pulmonary complications: (A) intervention type (perioperative ERAS vs. prehabilitation), (B) intervention provider (multidisciplinary team vs. nurse-led), and (C) study region. No between-subgroup difference was statistically significant.

Supplementary Figure S4.

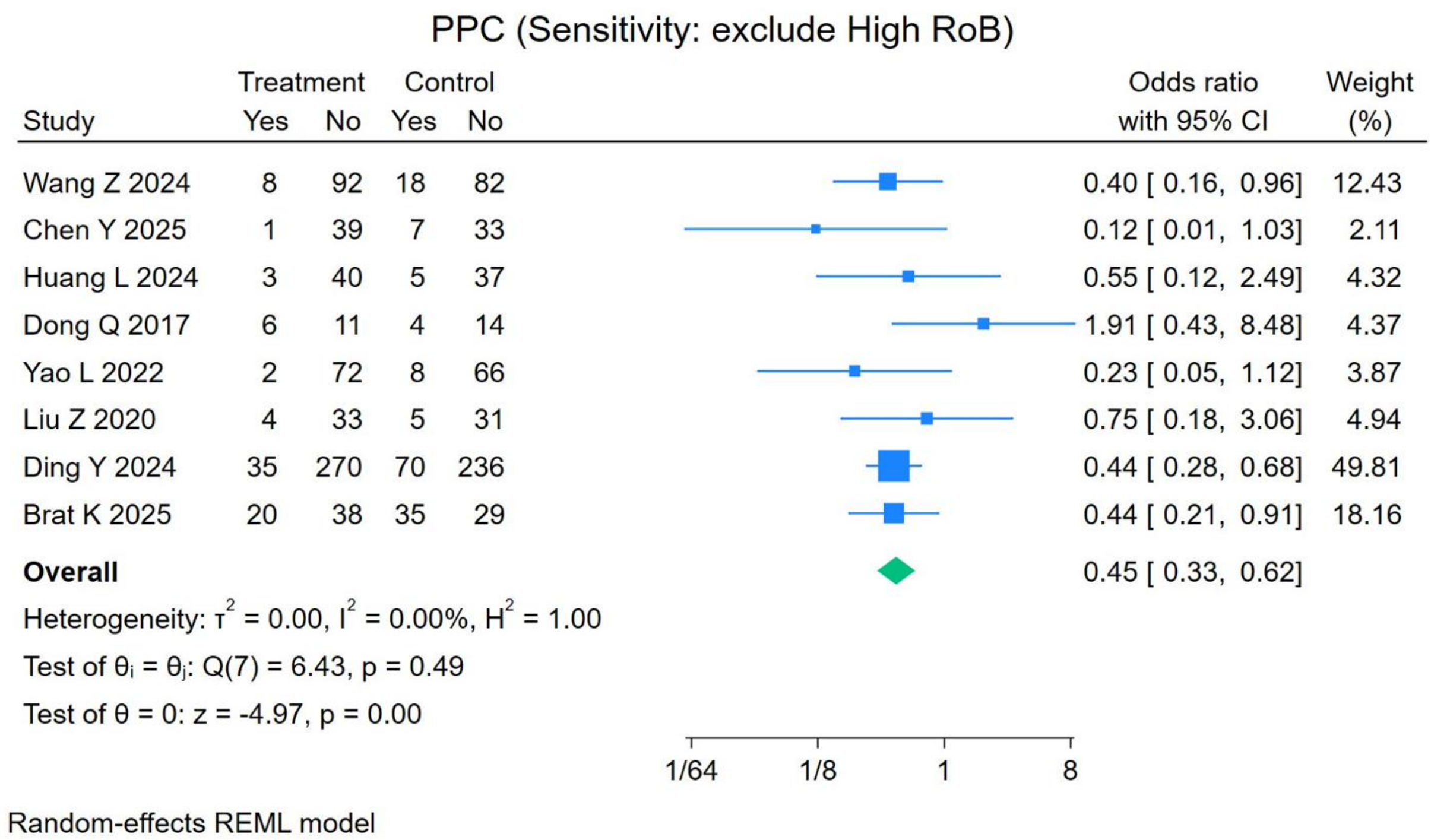

Sensitivity analysis for postoperative pulmonary complications excluding the study judged to be at high risk of bias (Qi F, 2025): OR 0.45 (95% CI 0.33–0.62), $I^2 = 0\%$.

Supplementary Figure S5.

(A)

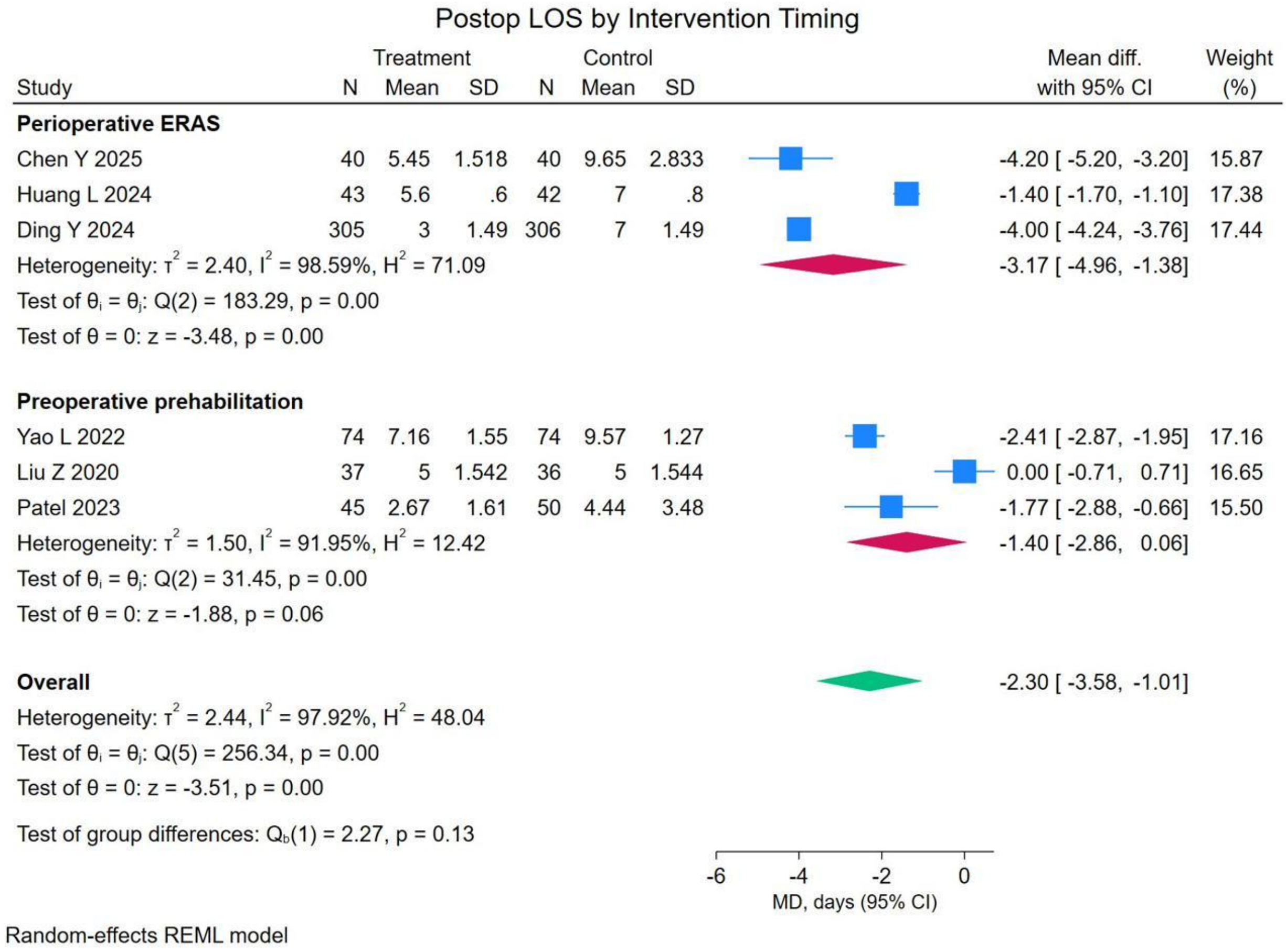

(B)

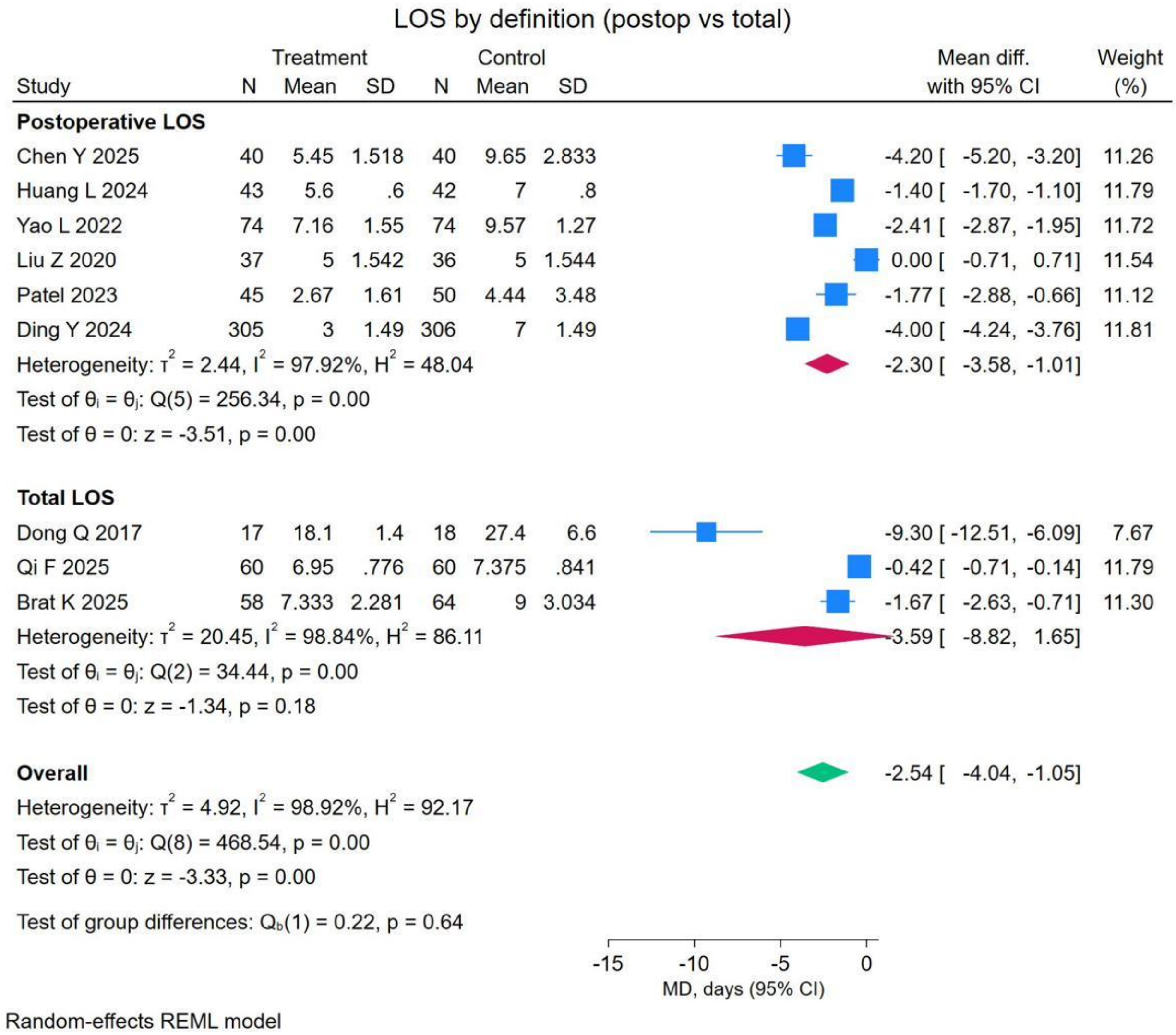

Subgroup analyses for length of hospital stay: (A) intervention type (perioperative ERAS vs. prehabilitation) and (B) definition of LOS (postoperative vs. total hospital stay). Neither between-subgroup difference was statistically significant.

Although the study protocol specified narrative synthesis when heterogeneity exceeded 75%,

Supplementary Figure S6.

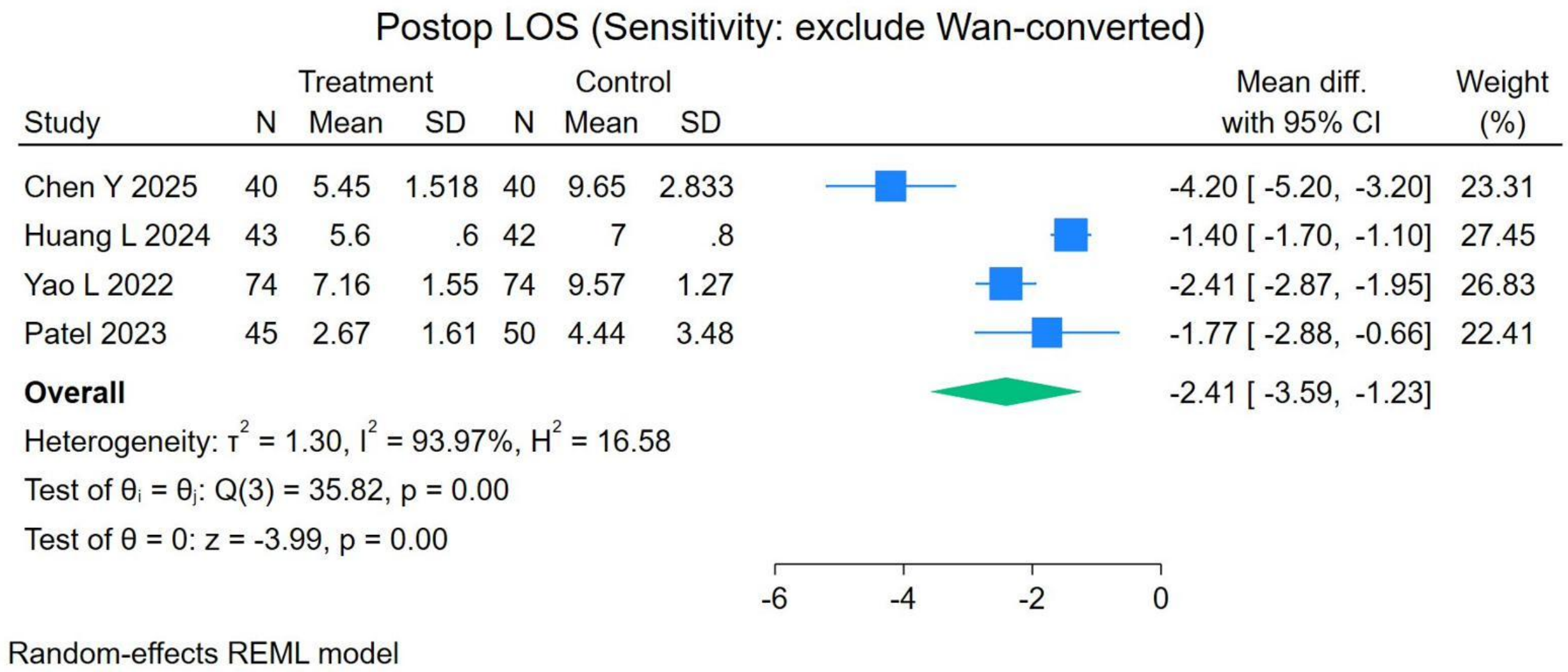

Sensitivity analysis for postoperative length of hospital stay excluding studies requiring statistical conversion of summary data (Wan et al. method): MD −2.41 days (95% CI −3.59 to −1.23).

Supplementary Figure S7.

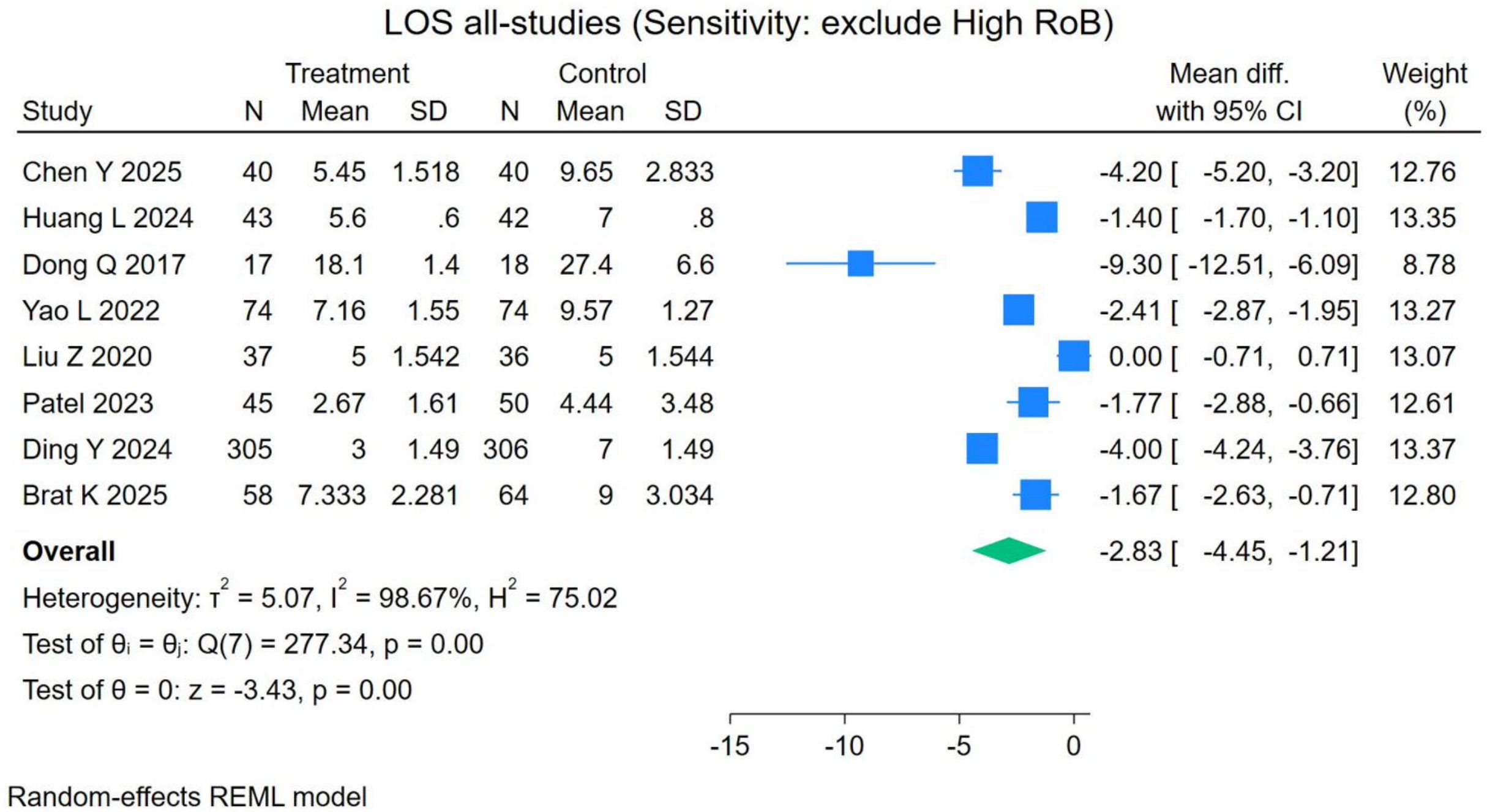

Sensitivity analysis for length of hospital stay excluding the study judged to be at high risk of bias: MD −2.83 days (95% CI −4.45 to −1.21).
